# Social Class and Changes in Australian Women's Affect and Alcohol Consumption During COVID-19

**DOI:** 10.3389/fpubh.2021.645376

**Published:** 2021-06-29

**Authors:** Belinda Lunnay, Barbara Toson, Carlene Wilson, Emma R. Miller, Samantha Beth Meyer, Ian N. Olver, Kristen Foley, Jessica A. Thomas, Paul Russell Ward

**Affiliations:** ^1^Department of Public Health, College of Medicine and Public Health, Flinders University, Bedford Park, SA, Australia; ^2^College of Medicine and Public Health, Flinders University, Bedford Park, SA, Australia; ^3^Olivia Newton-John Cancer Research Institute, Heidelberg, VIC, Australia; ^4^School of Psychology and Public Health, College of Science, Health, and Engineering, La Trobe University, Melbourne, VIC, Australia; ^5^School of Public Health and Health Systems, Faculty of Applied Health Sciences, University of Waterloo, Waterloo, ON, Canada; ^6^School of Psychology, Faculty of Health and Medical Sciences, University of Adelaide, Adelaide, SA, Australia

**Keywords:** alcohol, women, social class, survey, pandemic (COVID-19), anxiety, depression, uncertainty

## Abstract

**Introduction:** Before the pandemic, mid-life women in Australia were among the “heaviest” female alcohol consumers, giving rise to myriad preventable health risks. This paper uses an innovative model of social class within a sample of Australian women to describe changes in affective states and alcohol consumption patterns across two time points during COVID-19.

**Methods:** Survey data were collected from Australian mid-life women (45–64 years) at two time points during COVID-19—May 2020 (*N* = 1,218) and July 2020 (*N* = 799). We used a multi-dimensional model for measuring social class across three domains—economic capital (income, property and assets), social capital (social contacts and occupational prestige of those known socially), and cultural capital (level of participation in various cultural activities). Latent class analysis allowed comparisons across social classes to changes in affective states and alcohol consumption patterns reported at the two time points using alcohol consumption patterns as measured by the Alcohol Use Disorders Identification Test—Consumption (AUDIT-C) and its component items.

**Results:** Seven social classes were constructed, characterized by variations in access to capital. Affective states during COVID-19 differed according to social class. Comparing between the survey time points, feeling fearful/anxious was higher in those with high economic and cultural capital and moderate social capital (“emerging affluent”). Increased depression was most prominent in the class characterized by the highest volumes of all forms of capital (“established affluent”). The social class characterized by the least capital (“working class”) reported increased prevalence of uncertainty, but less so for feeling fearful or anxious, or depressed. Women's alcohol consumption patterns changed across time during the pandemic. The “new middle” class—a group characterized by high social capital (but contacts with low prestige) and minimal economic capital—had increased AUDIT-C scores.

**Conclusion:** Our data shows the pandemic impacted women's negative affective states, but not in uniform ways according to class. It may explain increases in alcohol consumption among women in the emerging affluent group who experienced increased feelings or fear and anxiety during the pandemic. This nuanced understanding of the vulnerabilities of sub-groups of women, in respect to negative affect and alcohol consumption can inform future pandemic policy responses designed to improve mental health and reduce the problematic use of alcohol. Designing pandemic responses segmented for specific audiences is also aided by our multi-dimensional analysis of social class, which uncovers intricate differences in affective states amongst sub-groups of mid-life women.

## Introduction

The COVID-19 pandemic has been disorientating and disruptive for many Australians. Although Federal and State governments have had success in controlling SARS-CoV-2 infection rates (1), the measures taken to suppress viral spread, including social distancing and lockdown restrictions, have had far reaching consequences. These include impacts on the economy and the ability to socialize at work, with friends, and through recreational and cultural activities. Some data suggest that, in Australia, the impact has been particularly pronounced for certain sub-sets of the population, particularly those who are already facing hardships or vulnerability (2, 3), with emerging evidence that this result is mimicked internationally (4, 5). The nuanced impacts of the pandemic, which likely differ between groups of women, require close examination. Differences in possible sequelae of the strategies implemented to reduce COVID-19 risk, including affective states (e.g., changes in feelings of fear, anxiety, or depression) and alcohol consumption, require identification because of their impact on population health; central to the present study, the physical and social costs of mental health decline and health risks associated with alcohol consumption. The nature of Australian women's alcohol consumption before the pandemic, and the unprecedented change in Australians' life circumstances evoked through COVID-19 suppression policies, need to be examined together because there is a possibility that the latter may impact adversely on the former.

Before the emergence of COVID-19, we had commenced a study designed to explore the role of alcohol in the lives of women from different social classes during the life stage defined as “mid-life” (45–64 years). Women in mid-life consume alcohol more than any other age group (6), despite the fact that high frequency drinking is associated with myriad acute and chronic health risks including liver disease, high blood pressure, overweight and obesity and cancer (7). For this reason, adults in mid-life are identified as a priority group in Australia's National Alcohol Strategy 2019–2028 (8). Early data from our study suggest that mid-life women consumed alcohol to release stress and also that women from different social class groups consume alcohol at different levels and for varying reasons, requiring different public health responses (9, 10). National data emerging during the COVID-19 pandemic showed that the frequency of alcohol consumption increased amongst Australian women (11). Of the sample surveyed (*n* = 561) 47.9 per cent of women self-reported an increase of 1–2 standard drinks of alcohol per week. These data also show alcohol consumption amongst women increased more than amongst men (22.8 per cent compared to 17.9 per cent).

Albeit this report was not designed to capture detail specific enough to develop targeted public health policy responses (e.g., by way of targeted messaging). However, psychological distress amongst Australians at the outset of the pandemic was found to be associated with increases in alcohol consumption (11). This is important, given we know that consuming alcohol is linked to broader environmental conditions; for example, the conditions leading to psychological distress and associated alcohol consumption are not uniform. As such, public health recommendations for—in this case, reducing consumption—is contingent on the “real possibilities” for target audiences (12). Women's affective states during the pandemic provide an important context to their sense of risk and specifically, the negative affect stemming from the impact of pandemic countermeasures (including various lockdowns and restrictions), and is likely discernible by social class—that is, by the resources and levels of advantage that shape women's daily living. Stress and isolation, as common reasons for women's alcohol consumption gleaned in our previous research are potentially inflated by the various environmental and commercial aspects of alcohol consumption during COVID-19 lockdowns that might make limiting alcohol difficult (13).

This paper describes differences in Australian women's affective states during COVID-19 and their alcohol consumption patterns according to social class. We interpret any change across two time points in affect and consumption and investigate women's experiences of living throughout times of the various restrictions put in place by the Government. Importantly, we have used a novel approach to operationalize social class that extends beyond simple economic, employment, and educational markers, recently used in the UK and Australia (14, 15) and is based on the seminal work of (16). This relational model has contemporary relevance to the nuances of social class divisions that extend beyond wealth to the social and cultural dimensions that shape life chances and health-related outcomes, thus offering advancement over previous influential measures for calculating social class (14). This model has particular utility for investigating diversity in responses to the pandemic because it emphasizes the “mutual constitution” (17) of economic and social facets in understanding the structuring of class and of inequalities, and is suitable for investigating the unintended consequences of countermeasures that manifest in tensions “between health and wealth” during the COVID-19 pandemic (18).

Data reported here were collected through an online survey as part of a broader national study of Australian women's alcohol consumption and their perceptions of the alcohol-related risk of breast cancer. The specific aim was to address the question, *does social class differentiate changes in alcohol consumption patterns and changes in affect during COVID-19?* To summarize, we tested the proposition that the impacts of the pandemic would be felt differently, in terms of change in affect and alcohol consumption patterns, by women in different social class groups in Australia.

## Methods

We conducted online surveys with mid-life women in Australia at two time points during COVID-19. A commercial panel was provided by Qualtrics (19) and a quota system was used by Qualtrics to recruit survey respondents who identified as female, were aged 45–64 years, initially recruiting for evenly distributed tertiles of household income, based on ABS definitions of “low,” “medium,” and “high” (20). Respondents with existing chronic conditions were excluded. A sample size of 600 was required to achieve a 4% margin of error with confidence intervals of 95%. To adjust for an expected 50% attrition between waves and ensure study power at time point 2, 1,200 respondents were required at time point 1.

The first survey was conducted in May 2020, not long after social distancing and various lockdowns and restrictions began in Australia^1^. A follow-up survey (with the same women) was undertaken 2 months later, in July 2020, when viral transmission was more controlled, infection rates reduced, and restrictions eased.

The survey comprised various items measuring general health status and risk perceptions, informing our broader study on mid-life women's alcohol consumption. Only those items that inform our analysis of the class-based differences in changes across time in feelings and alcohol consumption are reported in this paper; the others will be reported elsewhere. Herein, we report between social class group comparisons of changes between two time points during COVID-19 in terms of AUDIT-C (21) scores (an index of problematic alcohol consumption) and in changes in affect measured as yes or no responses for six positive and two negative affective states (explained in detail below).

Data were analyzed using Stata (version 16, Stata Corporation, College Station, TX, USA). Statistical patterns across social classes were examined using Kruskal-Wallis, Chi-square and Fisher's exact as appropriate.

### Survey Items

#### Demographic Measures

The survey items that inform this analysis include demographic information: age, relationship status, parenting status, living arrangements, the number of children living with them, education, household income and assets (property and savings), and post-code. Respondents reported their usual employment status and whether their work status or conditions had changed since the emergence of COVID-19.

#### Measures of “Capital” Used to Construct Social Class Categories

To construct social class groups, three forms of capital were measured (15). The questions replicate those utilized by Savage et al. in the 2011 Great British Class Survey to map class divisions in the UK (14). The survey tool was later reproduced by Australian researchers Sheppard and Biddle in 2015 to identify stratification in Australian society (15). Firstly, *Economic capital* was measured using household income and assets. Assets were measured by combining responses to the questions: *What is your annual income before tax or anything else is taken out?* (responses were indicated by income brackets provided); *What would you say is the approximate value of the property owned or mortgaged by you?* and *Roughly how much do you have in savings?* (< $20,000; $20,000 to <40,000, $40,000 to <60,000, $60,000 to <80,000, $80,000 to <100,000, $100,000 to <150,000 and $150,000 or more). Secondly, *Social capital* was measured by totalling the number of a range of known occupations within the respondent's social contacts (i.e., yes = 1) and the average prestige of those occupations. Occupational prestige was assigned using the Australian Socioeconomic Index 2006—a validated index for occupational prestige (22). Occupations included: secretary, nurse, teacher, cleaner, university lecturer, artist, electrician, office manager, solicitor, farm worker, chief executive, software designer, call center worker, and postal worker. Thirdly, *Cultural capital* was measured by a count of “highbrow” and “emerging” cultural activities (where 1 = yes), as per Bourdieu's description of cultural tastes. Respondents selected activities they had engaged in within the preceding 12 months from a list of possible cultural activities. The activities included: seen plays or gone to the theater, watched ballet or dance, gone to the opera, gone to museums or galleries, listened to jazz, listened to classical music (classified as “highbrow”) and listened to rock and/or indie music, attended gigs, played video games, watched sports, exercised or gone to the gym, used Facebook and/or Twitter, done arts and crafts, socialized at home, listened to rap music (classified as “emerging”).

#### Alcohol and Affect Measures

The survey also requested that respondents select from a list (yes or no) those feelings that applied to them “*during the COVID-19 pandemic*.” The exact question was: *Have you felt any of the following during the COVID-19 pandemic?* Response options were fearful/anxious, depressed, more connected with people (e.g., *via* social media or with neighbors/local community), isolated/lonely, hopeful about the future, a reduced sense of control, pessimism about the future, uncertainty.

Alcohol consumption patterns were measured using the 3-item Alcohol Use Disorder Identification Test—Consumption (AUDIT-C), which provides a total score out of 12 and allows determination of “problematic” alcohol consumption according to frequency and quantity consumed. The AUDIT-C tool has been validated for use in the general population (21, 23). An AUDIT-C score of 4 or above is considered indicative of problematic drinking (based on health and/or safety).

The second survey repeated only the alcohol questions and the measures of reactions to the pandemic. Respondents took 3–5 min to complete each survey.

### Ethics and Consent

The study was approved by the (redacted for review). The first page of each survey described the study in full including contact details for the research team and explaining that respondents had been invited to complete two surveys. Respondents provided consent by selecting “yes” to a series of conditions at the beginning of the survey, and for their de-identified responses to be used for research, per the Australian National Statement on Ethical Conduct of Human Research (24).

### Analytic Methods

**Figure 1:**
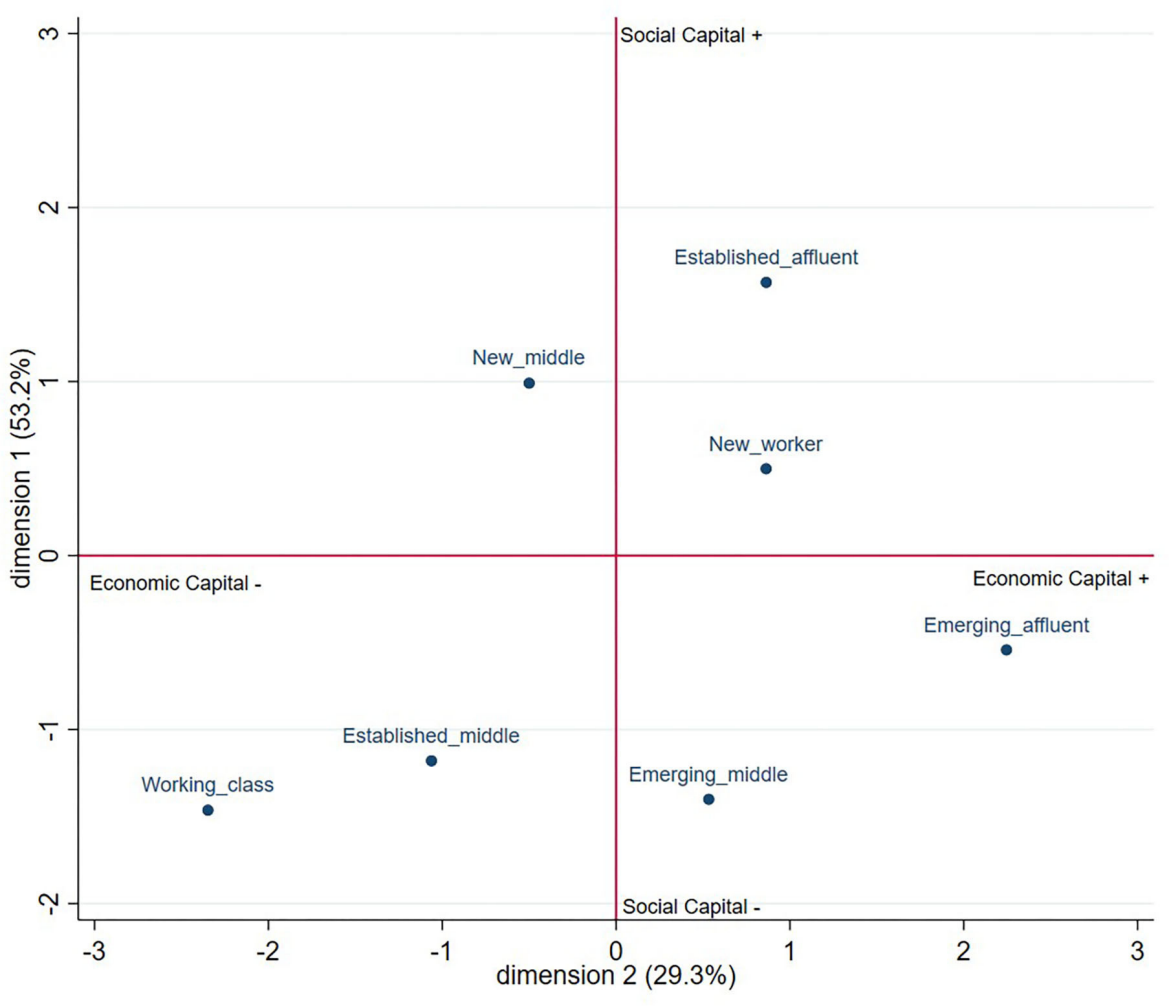
The position of social classes in social space by economic and social capital.

We analyzed data in several steps. We began with the outcome of Latent Class Analysis (LCA), outlining the different sub-groups of women distinguishable by marginal mean scores for the five measures indicating compositions of economic, social and cultural capital (see Table 1). We then described and labeled each of the social class groups based on responses at time point 1; *N* = 1,218 (Table 2). The social class groups were differentiated by their access to different compositions of capital and these are graphically depicted on two axes comprising economic (x axis) and social capital (y axis) (Figure 1). Once the social classes and the composition of capitals that characterize them were determined, we conducted Chi-square tests of independence, Fisher's exact-test or Kruskal-Wallis-test were performed as appropriate to explore the relation between social class and changes in affect and social class and AUDIT-C score. Change in responses across the two time points (*N* = 799 respondents completed both surveys) (see Table 3) was categorized into: no change, increase or decrease. To examine the relation between social class and the categorical variable indicating change across time for the variables of interest (affect and AUDIT-C score) a Chi square-test of independence was performed.

**Table 1:** Marginal means of the variables used in the LCA by class.

	**Economic capital**		**Social capital**		**Cultural capital**
**Class label**	**Household income**	**Assets (property and savings)**	**Known social contacts**	**Prestige of social contacts**	**Emerging cultural activities**
Working	1.89	1.50	1.23	1.36	2.17
New worker	2.58	1.71	3.72	4.23	2.84
Emerging middle	2.15	1.50	1.53	4.84	2.22
Established middle	3.02	4.31	1.31	1.42	2.40
New middle	2.17	1.56	3.90	2.56	2.48
Emerging affluent	3.40	4.29	2.58	4.73	2.30
Established affluent	3.55	4.37	4.36	3.40	3.05

**Table 2:** Summary of social classes resulting from LCA: labels and descriptions.

**Class label**	**Description**
Working	Members of the “working” class, comprising 22.9% of the sample (*n* = 279), have the **lowest of all forms of capital** and thus the **fewest resources and advantages** of all the classes. Members of this class report the *lowest income* and *fewest property and cash savings assets*. They also report the *fewest social contacts*, and their *known social contacts* are those with the *lowest occupational prestige*. Rates of educational attainment are lowest in the “working” class. Members comprising this class are the least likely of all classes to have completed University or College (20.8% reported completing University or College). They are the most likely of all classes to have achieved High/Secondary school as their highest level of education completed (53.4%) compared to other classes (which ranged from 18.2% of the “emerging affluent” class as the lowest to 36.4% of the “new middle” class as the next highest). Members of the “working” class had the *lowest participation in emerging cultural activities*. Members of this class are also most likely to be renting (30.3% of the overall sample) compared to other classes. This class are most likely to be living alone and most likely to be unemployed compared with other classes.
New worker	Members of the “new worker” class, comprising 12.3% of the sample (*n* = 151) have access to moderate income (more than the “working” class) but comparable to the “new middle” and the “emerging middle” classes, are low in property and savings assets (comparable to the “working” class). Social contacts amongst respondents comprising this class are higher than for the “working” class and not as high as scores for the “emerging middle” class. The occupational prestige of known contacts is higher than the scores for members of the “new middle” class. This class has the highest representation of respondents who reported having completed a trade certificate or apprenticeship (27.2%). Members of the “new worker” class are most likely to be working full-time than other classes.
Emerging middle	By comparison with the “working” and the “new worker” classes members of the “emerging middle” class, comprising 7.9% of the sample (*n* = 97), have more prestigious social networks (i.e., the score for occupational prestige of known contacts is higher than for the “working class”). Otherwise, members of the “emerging middle” class have access to similar amounts of economic resources as the “working” class, in fact they have slightly lower income than the “working” class but economic capital is comparable in terms of property and savings assets. Educational attainment amongst members of the “emerging middle” class is comparable to the “working” class, with members of the “emerging middle” class most likely to have completed Primary/Junior school but not have completed High/Secondary school. Unlike the “working” class, members of “emerging middle” class, while low in social contacts (like the “working” class), have social networks who work in occupations with high prestige.
Established middle	Members of the “established middle” class, comprising 9.6% of the sample (*n* = 118), report greater economic advantages (comparable the “emerging affluent” and “established affluent” classes) than the “working,” “new middle,” and “emerging middle” classes, but low social capital (comparable to the “working” class). This class is characterized by moderate levels of educational attainment. Members of the “established middle” class are most likely to be living with their partner and no children. They have a high representation of retirees.
New middle	Members of the “new middle” class, comprising 20.7% of the sample (*n* = 253), have low incomes and most comparable to the “emerging middle” class. They have more social contacts than members of the “emerging middle” class but their social contacts do not represent prestigious occupations like members the “emerging middle” class.
Emerging affluent	Members of the “emerging affluent” class, comprising 11.7% of the sample (*n* = 143), had amongst the highest income and assets (property and savings). Members of this class report low social contacts, but their known contacts represent occupations with high prestige. Members of the “emerging affluent” class were most likely to have completed University or College (67.8%). This class has the highest representation of retirees (along with members of the “established affluent” class) and students.
Established affluent	Members of the “established affluent” class, comprising 14.5% of the sample (*n* = 177) are the **most well-rounded in all forms of capital**. Overall, they have the **most resources and advantages** of all the classes. Members of this class are among those most likely to have completed University or College (60.5%) (comparable with the emerging affluent class). They have the highest participation in emerging cultural activities. Members of this class are most likely to be living with a partner and with children and a high representation of retirees.

**Table 3:** Respondent characteristics.

	**Working**	**New worker**	**Emerging middle**	**Established middle**	**New middle**	**Emerging affluent**	**Established affluent**
	***N* = 175**	***N* = 104**	***N* = 55**	***N* = 88**	***N* = 163**	***N* = 97**	***N* = 117**
Age, median (IQR)	55.0 (50.0, 60.0)	54.0 (48.0, 58.0)	55.0 (50.0, 60.0)	55.5 (50.0, 59.0)	54.0 (49.0, 59.0)	55.0 (50.0, 59.0)	53.0 (48.0, 59.0)
**Education level**							
Up to high/secondary school	101 (57.7%)	26 (25.0%)	19 (34.5%)	33 (37.5%)	61 (37.4%)	16 (16.5%)	36 (30.8%)
Trade certificate or apprenticeship	40 (22.9%)	30 (28.8%)	17 (30.9%)	22 (25.0%)	41 (25.2%)	15 (15.5%)	16 (13.7%)
University or college	34 (19.4%)	48 (46.2%)	19 (34.5%)	33 (37.5%)	61 (37.4%)	66 (68.0%)	65 (55.6%)
**Savings**							
< $20 k	114 (80.3%)	77 (79.4%)	34 (73.9%)	10 (12.8%)	102 (73.9%)	21 (24.1%)	22 (21.0%)
$20–40 k	13 (9.2%)	8 (8.2%)	5 (10.9%)	13 (16.7%)	17 (12.3%)	6 (6.9%)	22 (21.0%)
$40–$60 k	3 (2.1%)	5 (5.2%)	1 (2.2%)	7 (9.0%)	4 (2.9%)	17 (19.5%)	10 (9.5%)
$60–80 k	1 (0.7%)	0 (0.0%)	0 (0.0%)	4 (5.1%)	5 (3.6%)	6 (6.9%)	5 (4.8%)
$80–100 k	1 (0.7%)	0 (0.0%)	4 (8.7%)	9 (11.5%)	1 (0.7%)	12 (13.8%)	4 (3.8%)
$100–150 k	5 (3.5%)	0 (0.0%)	0 (0.0%)	4 (5.1%)	3 (2.2%)	9 (10.3%)	14 (13.3%)
$150 k plus	5 (3.5%)	7 (7.2%)	2 (4.3%)	31 (39.7%)	6 (4.3%)	16 (18.4%)	28 (26.7%)
**Property value**							
< $250 K	24 (21.6%)	10 (10.9%)	7 (20.0%)	0 (0.0%)	25 (18.2%)	0 (0.0%)	0 (0.0%)
$250–500 K	56 (50.5%)	34 (37.0%)	16 (45.7%)	0 (0.0%)	57 (41.6%)	1 (1.2%)	0 (0.0%)
$500–1 million	31 (27.9%)	47 (51.1%)	12 (34.3%)	38 (52.1%)	49 (35.8%)	37 (44.6%)	38 (36.5%)
1 million or more	0 (0.0%)	1 (1.1%)	0 (0.0%)	35 (47.9%)	6 (4.4%)	45 (54.2%)	66 (63.5%)
Renting	39 (26.0%)	7 (7.1%)	13 (27.1%)	7 (8.8%)	14 (9.3%)	8 (8.8%)	3 (2.8%)
**Household income**							
< $20,000	27 (15.7%)	5 (4.8%)	3 (5.5%)	2 (2.3%)	5 (3.1%)	0 (0.0%)	1 (0.9%)
$20,000 to <40,000	49 (28.5%)	8 (7.7%)	7 (12.7%)	9 (10.2%)	26 (16.0%)	3 (3.1%)	3 (2.6%)
$40,000 to <60,000	33 (19.2%)	23 (22.1%)	14 (25.5%)	5 (5.7%)	37 (22.7%)	5 (5.2%)	6 (5.1%)
$60,000 to <80,000	33 (19.2%)	14 (13.5%)	9 (16.4%)	5 (5.7%)	33 (20.2%)	13 (13.4%)	11 (9.4%)
$80,000 to <100,000	17 (9.9%)	23 (22.1%)	12 (21.8%)	14 (15.9%)	23 (14.1%)	19 (19.6%)	21 (17.9%)
$100,000 to <150,000	10 (5.8%)	23 (22.1%)	8 (14.5%)	27 (30.7%)	32 (19.6%)	35 (36.1%)	44 (37.6%)
$150,000 or more	3 (1.7%)	8 (7.7%)	2 (3.6%)	26 (29.5%)	7 (4.3%)	22 (22.7%)	31 (26.5%)
Living alone	47 (26.9%)	18 (17.3%)	6 (10.9%)	11 (12.5%)	32 (19.6%)	18 (18.6%)	10 (8.5%)
Number of children living with respondent, median (IQR)	2 (1, 2)	2 (1, 2)	1.5 (1, 2)	2 (1, 2)	2 (1, 2)	2 (1, 2)	2 (1, 2)
Paid work	83 (47.4%)	81 (77.9%)	38 (69.1%)	54 (61.4%)	118 (72.4%)	70 (72.2%)	92 (78.6%)
Full time work	38 (21.7%)	48 (46.2%)	19 (34.5%)	28 (31.8%)	53 (32.5%)	42 (43.3%)	45 (38.5%)

*Percentages may not total 100 due to rounding*.

#### LCA: Identifying Social Classes

We applied LCA to survey questions pertinent to the calculation of social class asked at time point 1. This approach allowed us to create social class groups that could be compared on their affective states and alcohol consumption patterns at each of the two survey time points during the COVID-19 pandemic. The number of classes was determined using both AIC (Akaike Information Criterion) and BIC (Bayesian Information Criterion). As described earlier, we adopted Sheppard and Biddle's (15) framework [a replication of Savage et al.'s study (14)] for determining social class, which they validated in Australia with a probability survey sample of 1,200 adults aged 18 years and over.

To ensure that the measures of capital had similar ranges while maintaining their distribution, they were transformed into quintiles before being entered into the final model. In our sample the variable associated with “highbrow” activities were highly skewed, and it was not possible to obtain quintiles and to include it in the LCA. Consequently, there was only one measure of cultural capital in our analysis (those considered to be “highbrow”), meaning not all types of capital had equal weighting in the final social class model. This is a point of difference with two previous studies that have used this framework (i.e., Sheppard and Biddle's study and that conducted by Savage et al.), in which the forms of capital have equal weighting in the final model.

Social classes are labeled and described based on the volume and composition of the various forms of capital characterizing each class by differences between the marginal means. Respondent's demographic details, specifically, education and living arrangements were included in the class descriptions as additional contextual information or “points of difference” where it helped to distinguish between groups.

Table 1 below indicates the marginal means for each of the seven social classes. These are then plotted in Figure 1 to illustrate different compositions of social and economic capital, and their “position in social space” relative to each other. Noting that participation in “emerging” cultural activities was about the same for all classes except for the “established affluent” class—members of this class reported slightly higher cultural capital.

Table 2 provides the social class labels and describes each class according to the composition of the various forms of capital that characterize the subgroup. Respondent's demographic details, specifically, education and living arrangements were included in the class descriptions as additional contextual information or “points of difference” where it helped to distinguish between groups. The descriptions provided in the social class models by Savage et al. (14) and Sheppard and Biddle's (15) models were guides.

## Results

Responses summarizing changes in affect and alcohol consumption and how these differ between social class groups at time point 1 are first provided (see Table 4) allowing for a “baseline.” We then present select results where change was observed between survey time point 1 and 2, differentiating type and prevalence of change by social class group (see Table 5). We detail only changes in affect variables that were statistically significant between social classes—that is, changes in feeling fearful or anxious, depression and uncertainty. We then outline changes in alcohol consumption patterns by social class, we offer some insight to the potential relationship between the reported changes by comparing the type of change (increase/decrease) in affect and change in alcohol consumption (increase/decrease in AUDIT-C scores), looking for patterns.

**Table 4:** Affect and alcohol patterns at time point 1 by social class (*n* = 799).

	**Working**	**New worker**	**Emerging middle**	**Established middle**	**New middle**	**Emerging affluent**	**Established affluent**	***p*-value**
Fearful or anxious								0.031
No	107 (61.1%)	58 (55.8%)	26 (47.3%)	64 (72.7%)	97 (59.5%)	65 (67.0%)	64 (54.7%)	
Yes	68 (38.9%)	46 (44.2%)	29 (52.7%)	24 (27.3%)	66 (40.5%)	32 (33.0%)	53 (45.3%)	
Depression								0.046
No	144 (82.3%)	79 (76.0%)	38 (69.1%)	76 (86.4%)	118 (72.4%)	75 (77.3%)	84 (71.8%)	
Yes	31 (17.7%)	25 (24.0%)	17 (30.9%)	12 (13.6%)	45 (27.6%)	22 (22.7%)	33 (28.2%)	
More connected with people								0.15
No	149 (85.1%)	84 (80.8%)	49 (89.1%)	80 (90.9%)	131 (80.4%)	79 (81.4%)	91 (77.8%)	
Yes	26 (14.9%)	20 (19.2%)	6 (10.9%)	8 (9.1%)	32 (19.6%)	18 (18.6%)	26 (22.2%)	
Isolated/lonely								0.33
No	128 (73.1%)	68 (65.4%)	37 (67.3%)	69 (78.4%)	108 (66.3%)	72 (74.2%)	82 (70.1%)	
Yes	47 (26.9%)	36 (34.6%)	18 (32.7%)	19 (21.6%)	55 (33.7%)	25 (25.8%)	35 (29.9%)	
Hopeful about the future								0.45
No	135 (77.1%)	80 (76.9%)	47 (85.5%)	67 (76.1%)	136 (83.4%)	76 (78.4%)	98 (83.8%)	
Yes	40 (22.9%)	24 (23.1%)	8 (14.5%)	21 (23.9%)	27 (16.6%)	21 (21.6%)	19 (16.2%)	
A reduced sense of control								0.007
No	135 (77.1%)	60 (57.7%)	32 (58.2%)	59 (67.0%)	115 (70.6%)	62 (63.9%)	71 (60.7%)	
Yes	40 (22.9%)	44 (42.3%)	23 (41.8%)	29 (33.0%)	48 (29.4%)	35 (36.1%)	46 (39.3%)	
Pessimism about the future								0.15
No	140 (80.0%)	70 (67.3%)	40 (72.7%)	67 (76.1%)	129 (79.1%)	73 (75.3%)	81 (69.2%)	
Yes	35 (20.0%)	34 (32.7%)	15 (27.3%)	21 (23.9%)	34 (20.9%)	24 (24.7%)	36 (30.8%)	
Uncertainty								<0.001
No	91 (52.0%)	32 (30.8%)	15 (27.3%)	40 (45.5%)	45 (27.6%)	37 (38.1%)	36 (30.8%)	
Yes	84 (48.0%)	72 (69.2%)	40 (72.7%)	48 (54.5%)	118 (72.4%)	60 (61.9%)	81 (69.2%)	
Total AUDIT-C score—wave 1, median (IQR)	3 (2, 5)	3 (2, 4)	3 (2, 4)	3 (2, 5)	3 (2, 5)	3 (1, 4)	3.5 (2, 5)	0.18

*Percentages may not total 100 due to rounding*.

**Table 5:** Changes in affect and AUDIT-C scores by social class group (*n* = 799).

	**Working**	**New worker**	**Emerging middle**	**Established middle**	**New middle**	**Emerging affluent**	**Established affluent**	***p*-value**
Fearful or anxious								0.007
Less	22 (12.6%)	10 (9.6%)	15 (27.3%)	10 (11.4%)	23 (14.1%)	9 (9.3%)	25 (21.4%)	
Stayed the same	124 (70.9%)	75 (72.1%)	33 (60.0%)	61 (69.3%)	125 (76.7%)	65 (67.0%)	74 (63.2%)	
More	29 (16.6%)	19 (18.3%)	7 (12.7%)	17 (19.3%)	15 (9.2%)	23 (23.7%)	18 (15.4%)	
Depression								0.049
Less	15 (8.6%)	9 (8.7%)	6 (10.9%)	4 (4.5%)	27 (16.6%)	13 (13.4%)	19 (16.2%)	
Stayed the same	140 (80.0%)	80 (76.9%)	42 (76.4%)	77 (87.5%)	120 (73.6%)	77 (79.4%)	79 (67.5%)	
More	20 (11.4%)	15 (14.4%)	7 (12.7%)	7 (8.0%)	16 (9.8%)	7 (7.2%)	19 (16.2%)	
More connected with people								0.66
Less	15 (8.6%)	14 (13.5%)	3 (5.5%)	6 (6.8%)	17 (10.4%)	11 (11.3%)	17 (14.5%)	
Stayed the same	143 (81.7%)	77 (74.0%)	44 (80.0%)	71 (80.7%)	129 (79.1%)	71 (73.2%)	87 (74.4%)	
More	17 (9.7%)	13 (12.5%)	8 (14.5%)	11 (12.5%)	17 (10.4%)	15 (15.5%)	13 (11.1%)	
Isolated/lonely								0.69
Less	22 (12.6%)	17 (16.3%)	6 (10.9%)	10 (11.4%)	16 (9.8%)	8 (8.2%)	13 (11.1%)	
Stayed the same	125 (71.4%)	67 (64.4%)	43 (78.2%)	68 (77.3%)	123 (75.5%)	77 (79.4%)	88 (75.2%)	
More	28 (16.0%)	20 (19.2%)	6 (10.9%)	10 (11.4%)	24 (14.7%)	12 (12.4%)	16 (13.7%)	
Hopeful about the future								0.051
Less	18 (10.3%)	15 (14.4%)	4 (7.3%)	7 (8.0%)	13 (8.0%)	15 (15.5%)	10 (8.5%)	
Stayed the same	144 (82.3%)	83 (79.8%)	47 (85.5%)	77 (87.5%)	124 (76.1%)	75 (77.3%)	94 (80.3%)	
More	13 (7.4%)	6 (5.8%)	4 (7.3%)	4 (4.5%)	26 (16.0%)	7 (7.2%)	13 (11.1%)	
A reduced sense of control								0.46
Less	19 (10.9%)	15 (14.4%)	8 (14.5%)	16 (18.2%)	23 (14.1%)	16 (16.5%)	20 (17.1%)	
Stayed the same	115 (65.7%)	69 (66.3%)	39 (70.9%)	55 (62.5%)	114 (69.9%)	71 (73.2%)	79 (67.5%)	
More	41 (23.4%)	20 (19.2%)	8 (14.5%)	17 (19.3%)	26 (16.0%)	10 (10.3%)	18 (15.4%)	
Pessimism about the future								0.26
Less	19 (10.9%)	20 (19.2%)	7 (12.7%)	11 (12.5%)	15 (9.2%)	11 (11.3%)	22 (18.8%)	
Stayed the same	132 (75.4%)	64 (61.5%)	42 (76.4%)	64 (72.7%)	119 (73.0%)	69 (71.1%)	74 (63.2%)	
More	24 (13.7%)	20 (19.2%)	6 (10.9%)	13 (14.8%)	29 (17.8%)	17 (17.5%)	21 (17.9%)	
Uncertainty								0.002
Less	20 (11.4%)	14 (13.5%)	4 (7.3%)	7 (8.0%)	36 (22.1%)	16 (16.5%)	27 (23.1%)	
Stayed the same	110 (62.9%)	72 (69.2%)	45 (81.8%)	66 (75.0%)	105 (64.4%)	65 (67.0%)	71 (60.7%)	
More	45 (25.7%)	18 (17.3%)	6 (10.9%)	15 (17.0%)	22 (13.5%)	16 (16.5%)	19 (16.2%)	
AUDIT-C score								0.030
Less	47 (35.9%)	28 (31.8%)	15 (34.9%)	26 (37.7%)	23 (17.8%)	19 (22.6%)	38 (35.2%)	
Stayed the same	45 (34.4%)	35 (39.8%)	18 (41.9%)	30 (43.5%)	56 (43.4%)	41 (48.8%)	41 (38.0%)	
More	39 (29.8%)	25 (28.4%)	10 (23.3%)	13 (18.8%)	50 (38.8%)	24 (28.6%)	29 (26.9%)	

*Percentages may not total 100 due to rounding*.

### Respondent Characteristics

A total of 799 (65%) respondents completed both surveys. The following results report responses from women who completed surveys at time points 1 and 2. Age, education and social class were not predictors that respondents of the first survey (time point 1) would complete the second survey (time point 2).

### Affect and Alcohol Patterns at Time Point 1 by Social Class

**Figure 2:**
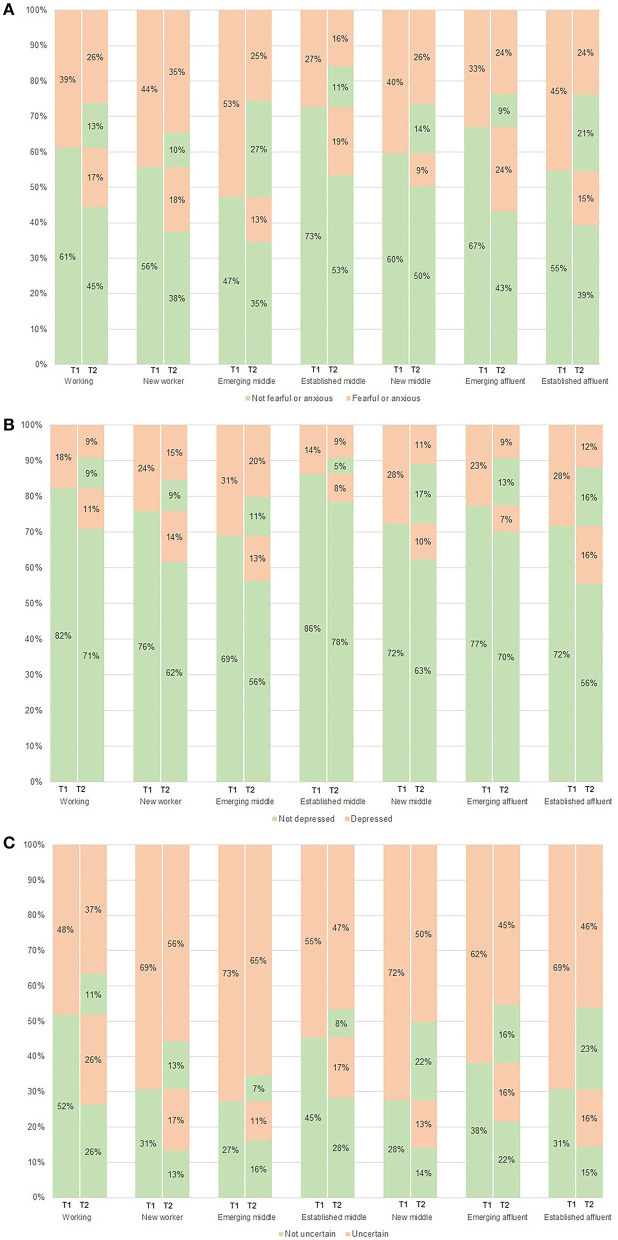
Changes in negative affect between time point 1 and time point 2 during COVID-19 by social class. **(A)** Feeling fearful or anxious. **(B)** Feeling depression. **(C)** Feeling uncertainty.

Table 4 and Figure 2 depict the changes in affect and alcohol consumption patterns at time point 1 (including only respondents who completed both surveys, *N* = 799). This is useful context for interpreting change between survey time points.

Statistically significant differences between social class groups at time point 1 were observable in four of the eight feelings—“fearful/anxious” “depression,” “uncertainty,” and “reduced sense of control” all reactions that show negative affect.

Table 4 shows that at time point 1, the “emerging middle” class was the most likely to respond “yes” to feeling fearful or anxious (52.7%) compared to other classes, particularly compared to the “established middle” class (27.3%). The “emerging middle” class was the most likely to report “yes” to feeling “uncertainty,” compared to the “working” class which was most likely to respond “no” to feeling uncertainty (52.0%). The “emerging middle” class was most likely to report “yes” to feeling depression (30.9%) and the “established middle” was the least likely (86.4%) to respond “yes” to feeling depression.

### Change in Affect Reactions and Pattern of Alcohol Consumption by Social Class

Changes in individual women's affect reactions and problematic alcohol consumption measured by AUDIT-C scores, observable as differences between social class groups, are reported in Table 5.

There were changes in women's responses yes/no, groupable by social class, to questions about feeling fearful or anxious and uncertainty during the pandemic—depicted in the lasagne plot below (see Figure 2). Figure 2 illustrates shifts from time point 1—May 2020 (T1) to time point 2—July 2020 (T2) (i.e., increases or decreases in women's response yes/no to feelings) with change illustrated by the percentage of the sample for each social class group (25).

Notable is that there was a statistically significant difference between social classes in response to feeling a reduced sense of control at time point 1—the “working” class was most likely to respond “no” (77.1%) to feeling a reduced sense of control. The “new worker” class was most likely (42.3%) to respond “yes” to feeling a reduced sense of control. However, there was no difference between social classes when we looked for changes at survey time points 1 and 2.

### Changes in Negative Affect During COVID-19 by Social Class

#### Changes in Feeling Fearful or Anxious Between Time Point 1 and Time Point 2 by Social Class

As shown in Table 5, changes in feeling fearful or anxious at time point 2 was largest in the “emerging affluent” class (23.7% of this class reported feeling “more” fearful or anxious at time point 2 than at time point 1). While only a small proportion of the “new middle” class reported feeling “more” fearful or anxious at time point 2 (9.2%), most “stayed the same” (76.7%), noting that nearly half of this class reported “yes” to feeling fearful or anxious at time point 1. Figure 2A shows that 40% of the “new middle” class continued to respond “yes” to feeling fearful or anxious.

As presented in Table 5, the “emerging middle” class was most likely to report feeling “less” fearful or anxious (27.3%). It is worth noting that at time point 1 just over half of this class (52.7%) reported “yes” to feeling fearful or anxious.

#### Changes in Feeling Depression Between Time Point 1 and Time Point 2 by Social Class

Table 5 shows the “established affluent” class was most likely to report feeling “more” depression (16.2%) more than any other social class. However, the “established affluent” class also reported feeling “less” depression (16.2%) alongside the “new middle” class (16.6%) more than any other class groups (noting that at time point 1, 28.2% of the “established affluent” class responded “yes” to feeling depression).

As shown in Table 4, the “established middle” class was the least likely to respond “yes” (13.6%) to feeling depression at time point 1 (86.4% responded “no” to feeling depression). Table 5 shows most of the “established middle” class reported they “stayed the same” in feeling depression (87.5%) while Figure 2B depicts that of the 86% who responded “no” to feeling depression at time point 1, 78% continued to report “no” at time point 2.

The “emerging affluent” class reported the lowest proportion of increase in feeling “more” depression (7.2%) at survey time point 2 (Table 5) and 22.7% responded “yes” to feeling depression at time point 1 (Table 4). Figure 2B shows that among those in this class reporting feeling depressed at point 1, more than half (around 57%) reported feeling less depressed at point 2 (13% of the total reporting no depression).

#### Changes in Feeling Uncertainty Between Time Point 1 and Time Point 2 by Social Class

As outlined in Table 5, the “working” class reported feeling “more” uncertainty (25.7%) the biggest increase reported at time point 2. Notable is that at time point 1, approximately half of this social class group (48.0%) responded “yes” to feeling uncertainty and 77% of these women continued to respond “yes” to feeling uncertainty at time point 2, representing 37% of the total 63% reporting depression—see Figure 2C.

The “emerging middle” class reported the least increase in feeling “more” uncertainty (10.9%) at time point 2. However, most of the “emerging middle” class “stayed the same” in feeling uncertainty (81.8%) this is notable given at time point 1 the “emerging middle” class reported the highest proportion who responded “yes” to feeling uncertainty (72.7%)—see Table 4. The large proportion of “emerging middle” class women who indicated they were feeling uncertain in both periods is clearly illustrated in Figure 2C, with ~89% remaining uncertain.

Similar to the “emerging middle” class, a large proportion of the “new middle” class responded “yes” to feeling uncertainty at time point 1 (72.4%)—see Table 4, and a large proportion “stayed the same” (64.4%) in reporting feeling uncertainty at survey time point 2—see Table 5. Figure 2C shows that ~69% of women reporting feeling uncertain at time point 1 also remained uncertain at point 2.

### Changes in AUDIT-C Scores by Social Class and Relationship Between Negative Affect and Change in Alcohol Consumption

The “new middle” class reported the lowest change in terms of a decrease in AUDIT-C scores (17.8% reported a lower AUDIT-C score at time point 2—see Table 5). The “established middle” class showed the largest proportion of decrease in AUDIT-C scores (37.7%) followed by the “working” (35.9%) and the “established affluent” (35.2%) classes. The “established middle” class was the most likely of the social classes to report “less” in terms of AUDIT-C score (37.7%) at survey time point 2—see Table 5. Almost half of the “emerging affluent” class (48.8%) reported alcohol consumption patterns at time point 2 that reflected no change (i.e., “stayed the same”) from time point 1.

For all classes, median AUDIT-C scores were 3 (IQ range 2–5) at time point 1, noting that a score of below 4 is considered low risk to health and safety. Exact McNemar's-tests determined that there were no statistically significant differences in the proportions of respondents scoring in the problematic drinking range (≥4) between surveys.

#### Alcohol Consumption and Feeling Fearful Or Anxious

The “new middle” class was the most likely to change AUDIT-C scores toward an increase. Table 5 shows 38.8% of this group reported increased scores and 40.5% of this group reported feeling fearful or anxious during COVID-19 at time point 1 with 76.7% reporting that their feelings in this regard remained the same at point 2.

The “emerging middle” class reported the most change in feeling “less” fearful or anxious at time point 2 (27.3%). Of the “emerging middle” class, 60% reported feeling the “same” level of fearfulness and anxiety, with the largest proportion of this group reporting feeling this way at time point 1−52.7%. A relatively large proportion of this group also reported a decrease in AUDIT-C score (for 34.9% scores were less) at time point 2—see Table 5. This result suggests that reduced feelings of fearfulness or anxiety might be associated with reduced alcohol consumption.

An interesting contrast is that respondents in the “emerging affluent” class, though most likely to report an increased prevalence of feeling fearful or anxious at survey time point 2 (23.7% said they felt “more” fearful or anxious), reported the second lowest change toward a reduced AUDIT-C score (22.6% reported “less” problematic alcohol consumption). The “new middle class” reported the lowest change with 17.8% reporting “less” problematic alcohol consumption.

Alcohol consumption and feeling fearful or anxious during the pandemic appear to be linked albeit with differential effects across social class groups.

#### Alcohol Consumption and Feeling Depression

Although the “established affluent” class was the most likely to have increased prevalence of feeling depression at time point 2 (16.2%) and more than half (67.5%) “stayed the same” (see Table 5), this did not seem to have a bearing on AUDIT-C scores (indicating problematic alcohol consumption). Results for the “established affluent” class showed this class either “stayed the same” (38.0%) or trended toward a decrease in alcohol consumption (for 35.2% scores were “less”).

The “emerging affluent” class was the least likely to report an increased prevalence of feeling depression at time point 2 (7.2%) and almost half of the “emerging affluent” class reported alcohol AUDIT-C scores at time point 2 that reflected no change (48.8% “stayed the same”). There does not appear to be a relationship between feeling depression and change in AUDIT-C scores for the “emerging affluent” class.

#### Alcohol Consumption and Feeling Uncertainty

The “new middle” class reported the largest proportion of increase in AUDIT-C scores (38.8%)—see Table 5. The “established middle” class showed the largest proportion of decrease in AUDIT-C scores (37.7%) followed by the “working” class (35.9%)—see Table 5. The “working” (25.7%), “new worker” (17.3%), and “established middle” (17.0%) classes experienced the most increase in feeling “more” uncertainty during COVID-19—see Table 5 and Figure 2. There did not appear to be a clear relationship between increased uncertainty and increased AUDIT-C score, but together these classes constituted the largest part of the increase in feeling uncertainty and also collectively contributed to the largest proportion of increase in AUDIT-C scores.

## Discussion

In this paper we address the question, *does social class differentiate change in affect and change in alcohol consumption patterns during COVID-19?* We tested the proposition that the impacts of the pandemic would be felt differently, in terms of change in affect and alcohol consumption patterns, by women in different social class groups in Australia. The various pandemic countermeasures experienced in Australia have placed restrictions on women's social life, with cultural and economic impacts that manifest in their differing affect reactions in our study. Our results underscore the salience of a complex model of social class that recognizes the interplay of economics, culture, and social aspects of opportunity that distinguish groups of people (26–28). This sophisticated model of social class has uncovered subtle nuances in women's affect reactions and alcohol consumption that would be otherwise unnoticed. Our results show very distinct differences in how particular groups of women (comprising the mid-life study population) reacted to COVID-19—in terms of affect states and alcohol consumption and we can identify groups who experienced the pandemic in more fraught and “problematic” ways. Setting our results within a social class framework, we can also interpret how affect reactions during the pandemic are moored in class distinctions, reflecting the symbolic dimensions of class characteristics. We found feeling more fearful or anxious was most prominent amongst women in the “emerging affluent” class (who reported feeling more fearful or anxious at timepoint 2 than any other class group). The “working” class was the most likely to experience an increase in feeling uncertainty. The “established affluent” class was the most likely to report an increase in feeling depression during COVID-19, while the “emerging affluent” and the “established middle” was the least likely.

A potential relationship between AUDIT-C score (problematic alcohol consumption) and negative affect between social classes observable through our study extends recent studies produced during COVID-19 that point to links between social distancing restrictions, increased mental health burden (3) and particularly relevant here, to increased frequency of alcohol consumption amongst Australian women (11). The “new middle” class who reported feeling fearful or anxious and reported the largest proportion of increase (and lowest change in terms of a decrease) in AUDIT-C scores points to a potential relationship between alcohol consumption and this negative affect reaction during COVID-19. There was no such relationship observed for problematic alcohol consumption and feeling uncertainty or depression. Although, we also know that not all women respond to crisis in the same way, and nor do they consume alcohol for the same reasons. From our previous research, we identified that mid-life women consume alcohol to cope with stress manifest in myriad forms (9, 10, 29), and pandemic countermeasures have increased the magnitude of stress in women's lives (30). This was echoed in our results presented herein, which show respondents in the “emerging affluent” class, for example, though most likely to report an increase in feeling fearful or anxious, reported unchanged AUDIT-C score between survey time points. To add further classed complexity, results from the “emerging middle” class suggests that reduced feelings of fearfulness or anxiety might be associated with reduced alcohol consumption. We have ascertained that the potential relationship between changes in negative affect and change in AUDIT-C scores during COVID-19 are not uniform for all Australian women comprising our sample.

The economic and social structure of the social world experienced an upheaval during COVID-19 lockdown restrictions impacting on work and employment conditions, with the potential to compromise one's “sense of place” in the world. This disruption compromises one's ability to practice what Durkheim referred to as “logical conformity” (31) in an effort to conserve social order. Using our study results, we can consider how the aspects of life that identify social classes, and women's sense of belonging within them, as well as class-based aspirations that distinguish social classes, might be jeopardized during the pandemic. Savage (32) explored the meanings of work and discussed the role that work or being employed takes in enhancing confidence, and to bolster class position—improving privilege and power. This sense of jeopardy perhaps exacerbates feelings of anxiety, fear, uncertainty, and depression, among some women more so than among others, depending on social class. For example, increased fear and anxiety during COVID-19 in our study population was observed most amongst the “emerging affluent” class. This class have the highest income and assets (property and savings)—comparable only to the “established affluent” class (high access to all types of capital)—of all social class groups. Women in this class are also highly educated, and although they report low social contacts, social networks extend to occupations with high occupational prestige. It could be that feeling fearful or anxious during COVID-19 is heightened for women in this social class through the risk of losing income or reduced asset values. The potential dismantling of the economic capital on which their social identity is being established, and restrictions on social life including limitations on forming new social relationships thus reducing opportunities for their existing (valuable) social capital to be used to improve social class status possibly perpetuates feelings of fear and anxiety. The COVID-19 crisis has shaped many aspects of women's everyday lived experience (30), with the potential to re-order social life as they knew it, and in doing so, disrupting their classed identities. We can interpret this in our results, which show that at the outset of the pandemic, half of the “emerging middle” class reported feeling fearful and anxious, and more than half said this stayed the same when surveyed during the pandemic. This class group, with low economic capital but high social capital characterized by social contacts with high levels of prestige, likely experienced limits on participating in the reassuring and symbolic dimensions of socializing—perhaps evoking anxiety as they found themselves descending into tedium and fear. To summarize, our results demonstrate that though different women comprising the mid-life study population all experienced fear and anxiety, the underpinning was not uniform across social groups—rather it was discernible by social class.

Of those we surveyed, women in the “established affluent” class increased prevalence of feeling more depression than any other social class. Notably this class also decreased prevalence of feeling depression more than any other class group (similarly to the “new middle” class). This suggests the temporal aspects of living through the pandemic, and perhaps as time went on, an increasing sense of risk and limitations of the pandemic, might have impacted on an increasing negative “depressive” affect within members of this group. Recent research suggests comparative optimism about COVID-19 (perceived risk of infection and recovery) is weaker during uncontrollable events (33). Responsibility is situated with individuals during COVID-19 distancing measures in Australia—the efficacy of public health measures relies on individual choices to “stay at home” and “do the right thing” and there is a moral significance to class theorized in social class literature (34). Combined, this suggests that different levels of responsibility would be inordinately felt by women in different social class positions during COVID-19. Perhaps the weight of moral responsibility imbued in their class identity lead the “established affluent” to report feeling more depression during the pandemic. This is interesting given the “established middle” class and the “emerging affluent” class were the most likely to report feeling less depression during the pandemic. Perhaps women in these groups had simply experienced “crisis fatigue” (35) and had “brought down their shutters” (35) resulting from the omnipresence of fear and uncertainty, displaying a level of acceptance of having no control (36). Albeit, the “established middle” class also had the amongst the highest representation of retirees, for whom being at home during lockdown restrictions meant no changes to work routines and might not be all that different to previous life.

With respect to feeling uncertainty during COVID-19, the “working” class experienced the largest increase. It is unsurprising that the “working” class were identified as experiencing uncertainty during the pandemic, given the reported lowest income and fewest property and cash savings/assets among women in this class group. Though we do not know specific job titles, low wage work is often precarious work particularly during pandemic conditions, with leave unpaid and little to no job security. This, coupled with poor access to resources *via* social contacts, might account for the highest level of uncertainty in this social class (37). Shilling and Mellor (38) describe conditions of “future-oriented reflexivity” with relevance to understanding the preconditions of uncertainty. They explain that being “future oriented” and having the ability to foresee and adopt to situations with new patterns of action are limited by structural factors as well as by agency. It is entirely possible that structural determinants shaped by social class impact the ability for women in the “working” class to be future oriented in turn increasing feelings of uncertainty during COVID-19.

## Strengths and Limitations

A key strength of our study is the social nuances between groups of mid-life women that our model of class illuminates by capturing diversity in reactions and behavior during COVID-19. These would be otherwise undetected if interpreted at a whole of population level. Women who shared similar social “space” in terms of their class characteristics relative to those possessed by other women, likely also share pandemic experiences, but these are subtle and would be difficult to detect if only economic or education were used as predictors of outcomes.

There are several limitations with the method employed for our study. In terms of the replicability of our social class model, our measure of cultural capital excluded high-brow cultural activities meaning not all capitals had equal weighting in the final model. This is a point of difference to the social class model used by Sheppard and Biddle (15) and is a potential theoretical limitation of our analysis. With respect to our sample, there are several limitations. Due to the nature of our online survey (per social distancing), our sample does not encompass Australian women who do not access digital technologies. We also did not sample for Aboriginal and Torres Strait Islander or ethnic minority groups, groups identified as particularly vulnerable to the health effects and, social/economic impacts of COVID-19. Also, the sample size comprising each of the social class groups (~200 per group) was too small to determine if statistical changes observed within social classes in negative affect correlated with changes in alcohol consumption.

## Conclusion

Comparisons in three negative reactions (both increases and decreases between time points) for “fearful / anxious,” “uncertainty,” and “depressed” and patterns of alcohol consumption between social class groups of women at two time points during COVID-19 are provided herein. Our findings identify where particular attention should be paid in future public health responses, toward certain sub-populations of women likely to fare worst through the pandemic. Our work has relevance for designing future public health responses to COVID-19 and into recovery phases of the pandemic segmented by population groups. Our study shows this sophisticated, multi-dimensional model has substantial advantage over less dynamic ways of interpreting disadvantage in pandemic outcomes based on unidimensional measures such as education or income alone. Material inequality is entirely relevant to COVID-19, and the social determinants of health shaping COVID-19 disparities warrants identification as far as disease transmission and economic impacts are concerned (5). However, class formations situated in the social and cultural aspects of opportunity are also tremendously relevant, given the nature and magnitude of social disruption as a by-product of pandemic countermeasures in Australia, and the flow on effect for adverse coping behaviors, like alcohol consumption. We can link such differences between groups of mid-life women to the characteristics manifest in their social class position within our broader population sample—to differences in life chances represented in compositions of economic, social and cultural capital and to the values embedded in their social class conditions.

## Data Availability Statement

The raw data supporting the conclusions of this article will be made available by the authors, without undue reservation.

## Ethics Statement

The studies involving human participants were reviewed and approved by Flinders University Social and Behavioral Research Ethics Committee. The patients/participants provided their written informed consent to participate in this study.

## Author Contributions

EM, PW, CW, and BL designed and conducted the study. BT, BL, EM, and CW analyzed the data. All authors contributed to writing the manuscript.

## Conflict of Interest

The authors declare that the research was conducted in the absence of any commercial or financial relationships that could be construed as a potential conflict of interest.

## Footnotes

^1^Available online at: https://medium.com/@deborahalupton/timeline-of-covid-19-in-australia-1f7df6ca5f23 (Note: COVID-19 was declared a pandemic on March 11, 2020).
